# Interactions between coral propagules in aquarium and field conditions

**DOI:** 10.1038/s41598-020-77557-2

**Published:** 2020-11-25

**Authors:** Poh Leong Loo, Anqi Li, Koh Siang Tan

**Affiliations:** 1 St. John’s Island National Marine Laboratory, Tropical Marine Science Institute, National University of Singapore, 18 Kent Ridge Road, Singapore, 119227 Singapore; 2 Department of Biological Sciences, Faculty of Science, National University of Singapore, Block S3 #05-01, 16 Science Drive 4, Singapore, 117558 Singapore

**Keywords:** Environmental impact, Ecology, Environmental sciences

## Abstract

The effects of intraspecific and interspecific interactions between three species of scleractinian coral micro-colonies, namely *Lithophyllon undulatum*, *Turbinaria mesenterina* and *Platygyra sinensis* were evaluated for their survivorship, tissue loss and growth in both field (in-situ) and aquarium (ex-situ) conditions over 12 weeks. Regardless of environmental conditions and interactions, *L. undulatum* survived better (91.7 ± 6.2%) than *T. mesenterina* (75.0 ± 25.0%) and *P. sinensis* (60.4 ± 39.5%). Similarly, *L. undulatum* registered the lowest tissue loss (0.5 ± 0.7%) as compared to *T. mesenterina* (14.3 ± 19.4%) and *P. sinensis* (22.0 ± 30.0%). However, *P. sinensis* gained more weight (3.2 ± 5.2 g) than either *T. mesenterina* (2.7 ± 2.4 g) or *L. undulatum* (0.8 ± 1.1 g). In both environments, all three species in intraspecific interaction generally had higher survivorship, lower tissue loss and better growth than those in interspecific interaction except the latter in in-situ conditions had a twofold increase in growth (5.8 ± 3.7 g) than the former in-situ conditions (2.8 ± 3.7 g). Hence, all three species are potentially suitable for transplantation and mariculture except perhaps for *P. sinensis* which performed poorly in ex-situ conditions. Corals can be transplanted either with different colonies of the same species or together with other coral taxa. This study demonstrated that *L. undulatum* should be transplanted between *T. mesenterina* and *P. sinensis* for optimal growth and survival.

## Introduction

Coastal modification in Singapore and in other tropical Southeast Asian countries has severely impacted local coral reefs. Coral reefs are ecologically and economically important as they not only support marine biodiversity but also provide various services including coastline protection, natural products leading to drug discovery, fisheries, tourism and recreation^1^. Singapore has lost more than 70% of its natural coral reef cover, mainly due to rapid coastal development^2^ and climate change^3^. Reclaimed land resulting from such activities is often protected from erosion, tidal surge and sea-level rise by seawalls and rocky slopes. Indeed, some 60% of Singapore’s coastline is now lined by artificial seawalls and this percentage is expected to increase further by two-fold from the current 314 km by 2030^2^.

Although the primary function of seawalls is to protect the newly created reclaimed land from the sea, they also inadvertently serve as an alternative habitat for many marine organisms. Corals found on seawalls are often the same species occurring on natural rocky shores and coral reefs, even though the diversity and abundance of coral species on seawalls are usually lower than that in natural habitats^4^. Compared to 56 genera of corals occurring on natural coral reefs in Singapore, only 17 genera were found on seawalls^4^. The densities of coral colonies are also lower in the intertidal zone^4^. Lack of structural complexity may be one reason why seawalls cannot support high biodiversity. Despite this limitation, common and even rare coral species are found on seawalls^4^. Hence, seawalls have the potential to alleviate biodiversity loss caused by past and future coastline modification and serve as viable alternative reef habitats for corals and other reef inhabitants.

Coral transplantation has a long history and is used extensively to facilitate reef restoration and rehabilitation^5^. It has also been tried out in Singapore to enhance the abundance and diversity of coral species on seawalls^3^, and has met with some success on a small scale, showing that transplantation is a feasible way to increase ecological value of seawalls, particularly in the intertidal zone^6^. In order to increase habitat complexity and species diversity on intertidal seawalls, transplantation of multiple coral species with different growth forms would be desirable. Nevertheless, our understanding of biological and ecological interactions on tropical seawalls is still at an infant stage. Corals on the intertidal zone of seawalls are subjected to greater environmental stress as compared to those in the subtidal zone. Thus, the suitability of coral species for transplantation onto seawalls and their competitive interactions amongst and between different coral taxa need to be evaluated prior to transplantation, to ensure good survivorship and growth particularly in the intertidal zone.

Despite intensive research to assess the outcomes of interspecific competitive interactions between corals and other benthic marine invertebrates such as turf algae, macroalgae, crustose coralline algae (CCA), bryozoans, tunicates and sponges^7–10^, spatial competition between and within coral genera and genotypes of the same species is still poorly defined and obscure. Spatial competition is a major factor determining community structure in diversity-rich ecosystems such as rainforests and coral reefs^11^, as space is always a main limiting resource for sessile clonal organisms residing in hard-substratum environments^12,13^. Additionally, spatial competition amongst scleractinian corals can influence the patterns of coral abundance and diversity^14^. Numerous field surveys have shown that competitive interactions of reef cnidarians involve complex and circular networks of competitive dominance amongst coral taxa^15,16^. Competition affects coral fitness i.e., reproduction^17^, survivorship^12^ and growth^18^. Competition generally has negative consequences for the overall fitness of corals^18,19^. However, corals are able to utilize and sustain a multi-dimensional space simultaneously (1) in the water column through vertical augmentation and (2) on the reef substratum, via (a) planar growth, (b) pivots on growth rate, (c) surrounding environmental factors, (d) aggression ability and (e) coral growth forms^11^. Intra- and inter competitive relationships amongst coral species can be either “indirect” via allelopathy and overtopping or “direct” by physical overgrowth and digestive activity^11^. The complexity of interaction outcomes between competing sessile coelenterates is the combined effect of a multitude of aggressive retaliations e.g., overgrowth, discharge of nematocyst, a “retreat growth” phenomenon, abnormal growth morphologies and border line formation between alien tissues^19^. The results of competition can also be influenced by anthropogenic disturbances^20^, size of the contesting colonies^21^, age class^22^ and increased demographic density that may result in greater mortality, decreased growth, lower reproduction and/or low recruitment^23,24^.

Long-term spatial competition could have a detrimental effect on reef biodiversity and population dynamics^20^. The competitive strategy deployed in any interaction is affected by the surrounding environmental conditions and specific life history idiosyncrasies^14^. For instance, slow-growing massive corals use digestive mechanisms, whilst fast-growing branching corals such as pocilloporids and acroporids employ overgrowth and physical overtopping^11^. These two growth morphologies can coexist as the aggressive nature of massive species is levelled by the rate at which branching species proliferate^25^. The competitive hierarchy of coral taxa is generally linked to aggression ability and varies geographically^26^. Alteration of environmental conditions can also define the outcomes of competitive interactions between and within coral taxa^27^, coinciding with the growing evidence of evolving coral taxa interactions associated with climate change in marine environments^28^. For instance, thermal stress decreased the competitive advantage of certain corals, resulting in the change of the composition of coral genera^29^.

This study investigated the effects of intraspecific and interspecific interactions between three species of coral micro-colonies, namely *Lithophyllon undulatum* (a locally rare species)*, Turbinaria mesenterina* (less common species) and *Platygyra sinensis* (common species) in relation to their survivorship, tissue loss and growth under ex-situ and in-situ environmental conditions. The objectives of this study were (1) to determine the suitability of the abovementioned species for mariculture and transplantation onto seawalls, and (2) to evaluate the viability of ex-situ nursery for coral rehabilitation and restoration.

## Results

### Water quality

Key parameters for all treatments tested in both in-situ and ex-situ environments were relatively similar throughout the experimental period of three months (*p* > 0.05). Salinity, temperature, dissolved oxygen (DO) and pH in both environments ranged from 32.17–33.18 psu, 29.80–29.86 °C, 8.78–9.49 mg L^−1^ and 8.43–8.46, respectively. Nevertheless, the DO concentrations in ex-situ conditions were slightly higher than that of in in-situ conditions (Table 1).

**Table 1:** Comparison of key environmental parameters in the field (in-situ) and aquarium (ex-situ) culture over a period of 3 months (weeks 0–12) (mean ± S.D.; n = 13).

Environmental parameters	In-situ conditions (treatments 1 and 2)	Ex-situ conditions (treatments 3 and 4)	Ex-situ conditions (treatment 5—control)	*p*-value
Salinity (psu)	33.18 ± 1.92	32.17 ± 1.88	32.29 ± 1.85	Kruskal–Wallis chi-squared value = 1.14, df = 2, *p* = 0.564
Temperature (°C)	29.86 ± 0.15	29.85 ± 0.31	29.80 ± 0.40	ANOVA, *F* (2,0.029) = 0.15, *p* = 0.859
DO (mg L^−1^)	8.78 ± 0.92	9.22 ± 1.12	9.49 ± 1.05	Kruskal–Wallis chi-squared value = 3.78, df = 2, *p* = 0.151
pH	8.46 ± 0.05	8.44 ± 0.05	8.43 ± 0.06	ANOVA, *F* (2,0.007) = 1.06, *p* = 0.359

### Survivorship of coral micro-colonies in intraspecific and interspecific interactions

Micro-colonies from different parent colonies of *Lithophyllon undulatum* (Lit) and *Turbinaria mesenterina* (Tur) survived well regardless of culture conditions (Table 2). However, half of *Platygyra sinensis* (Pla) micro-colonies died in ex-situ conditions whereas 12.5% did not survive under in-situ conditions. Similarly, the results of the interspecific interaction experiment showed that Lit was able to survive well with Tur and Pla as these three species survived regardless of culture conditions over the course of three months (Table 2)*.* In contrast, Pla survived well in in-situ conditions, but showed high mortality (87.5%) in ex-situ conditions regardless of species that they interacted with. Additionally, Tur sustained the highest mortality (62.5%) when they were placed with Pla in the field as compared to those in the aquarium (25.0%).

**Table 2:** The probability of survivorship (mean ± S.D. %; n = 8) of coral species under intraspecific and interspecific interactions in ex-situ and in-situ conditions.

Interaction	Interspecific						Intraspecific		
Coral micro-colonies	**Pla**-Lit	**Pla**-Tur	**Lit**-Pla	**Lit**-Tur	**Tur**-Pla	**Tur**-Lit	**Pla**-Pla	**Lit**-Lit	**Tur**-Tur
Ex-situ environment	12.50 ± 0.35	12.50 ± 0.35	87.50 ± 0.35	87.50 ± 0.35	75.00 ± 0.46	50.00 ± 0.53	50.00 ± 0.53	100.00 ± 0.00	100.00 ± 0.00
In-situ environment	100.00 ± 0.00	100.00 ± 0.00	87.50 ± 0.35	87.50 ± 0.35	37.50 ± 0.52	87.50 ± 0.35	87.50 ± 0.35	100.00 ± 0.00	100.00 ± 0.00

“Pla-Tur” denotes *Platygyra sinensis* micro-colonies were placed 1 cm apart from *Turbinaria mesenterina* micro-colonies.

Pla = *Platygyra sinensis*, Tur = *Turbinaria mesenterina*, Lit = *Lithophyllon undulatum.*

Bold indicates the coral species whose micro-colonies were examined for survivorship.

### Tissue loss

**Figure 1:**
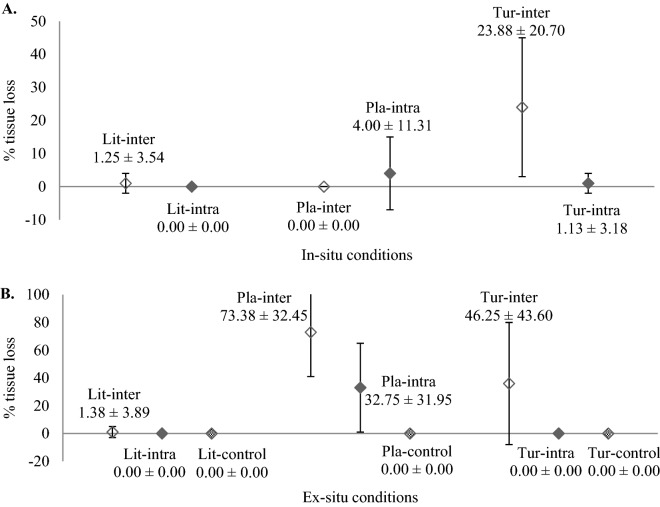
(**A**) Per cent tissue loss (mean ± S.D.; n = 8) of micro-colonies of three coral species after 3 months during which they were either subjected to intra- or inter-specific interactions under in-situ conditions. (**B**) Per cent tissue loss (mean ± S.D.; all treatments: n = 8 except control treatment: n = 16) of micro-colonies of three coral species after 3 months during which they were either subjected to intra- or inter-specific interactions and no interaction (control) under ex-situ conditions. Lit-inter = *Lithophyllon undulatum* in interspecific interaction; Lit-intra = *Lithophyllon undulatum* in intraspecific interaction; Lit-control = *Lithophyllon undulatum* without interaction; Pla-inter = *Platygyra sinensis* in interspecific interaction; Pla-intra = *Platygyra sinensis* in intraspecific interaction; Pla-control = *Platygyra sinensis* without interaction; Tur-inter = *Turbinaria mesenterina* in interspecific interaction; Tur-intra = *Turbinaria mesenterina* in intraspecific interaction; Tur-control = *Turbinaria mesenterina* without interaction.

Under in-situ culture conditions, only Tur had significantly higher (Kruskal–Wallis chi-squared value = 4.37, df = 1, *p* = 0.037) percentage of tissue loss incurred for interspecific interaction as compared to those subjected to intraspecific interaction (Fig. 1A). In contrast, Lit and Pla incurred either no or low tissue loss and no significant differences were observed between intra- and interspecies interactions (Fig. 1A). On the other hand, under ex-situ conditions, a significant difference in the percentage of tissue loss of Tur was observed between three treatments, where micro-colonies subjected to interspecific treatment suffered the highest tissue loss, whilst the other two treatments (i.e., intraspecific and control) had no tissue loss (Fig. 1B). Lit showed either little or no tissue loss across all three treatments (Fig. 1B). In contrast, percentage of tissue loss in Pla was significantly different amongst three treatments (Kruskal–Wallis chi-squared value = 21.67, df = 2, *p* = 1.969e−05). Highest percentage of tissue loss was recorded for micro-colonies subjected to interspecific interaction, whilst the lowest percentage of tissue loss was observed in the control treatment. Additionally, unlike Lit and Tur which had similar percentage of tissue loss amongst in-situ and ex-situ conditions, both intra- and interspecific treatments of Pla showed significantly lower percentage of tissue loss under in-situ conditions (intraspecific: Kruskal–Wallis chi-squared value = 5.37, df = 1, *p* = 0.021; interspecific: ANOVA, *F* (1,0.212) = 44.10, *p* = 1.11e−05) as compared to those under ex-situ conditions (Fig. 1A,B).

### Weight gain

**Figure 2:**
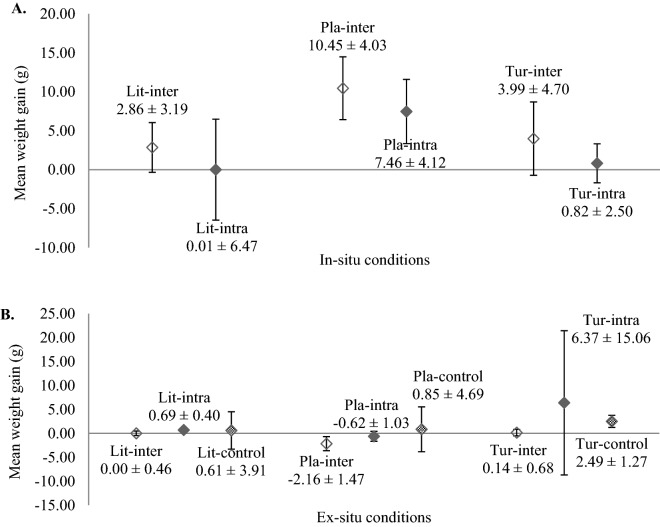
(**A**) Weight gain (mean ± S.D.; n = 8) of micro-colonies of three coral species after 3 months during which they were either subjected to intra- and inter-specific interactions under in-situ conditions. (**B**) Weight gain (mean ± S.D.; all treatments: n = 8 except control treatment: n = 16) of micro-colonies of three coral species after 3 months during which they were either subjected to intra- and inter-specific interactions or no interaction (control) under ex-situ conditions. Lit-inter = *Lithophyllon undulatum* in interspecific interaction; Lit-intra = *Lithophyllon undulatum* in intraspecific interaction; Lit-control = *Lithophyllon undulatum* without interaction; Pla-inter = *Platygyra sinensis* in interspecific interaction; Pla-intra = *Platygyra sinensis* in intraspecific interaction; Pla-control = *Platygyra sinensis* without interaction; Tur-inter = *Turbinaria mesenterina* in interspecific interaction; Tur-intra = *Turbinaria mesenterina* in intraspecific interaction; Tur-control = *Turbinaria mesenterina* without interaction.

Overall, weight gain of micro-colonies in the field under in-situ conditions was higher than in the aquarium subjected to ex-situ conditions, except for intraspecific interactions amongst Lit and Tur (Fig. 2A,B). Under in-situ culture conditions, micro-colonies of all three coral species tested for interspecific interaction had comparable mean weight gains with those subjected to intraspecific interaction (Pla: ANOVA, *F* (1,0.004) = 2.15, *p* = 0.165; Lit: ANOVA, *F* (1,0.001) = 0.50, *p* = 0.491; and Tur: ANOVA, *F* (1,0.005) = 2.83, *p* = 0.114) (Fig. 2A). Similarly, micro-colonies of Pla and Lit in three treatments cultured in ex-situ conditions were no significantly difference (Pla: ANOVA, *F* (2,0.003) = 2.03, *p* = 0.149; Lit: ANOVA, *F* (2,3.544e−05) = 2.05, *p* = 0.147) in terms of weight gain but the mean weights of micro-colonies subjected to intraspecific and control treatments were slightly higher than those subjected to interspecific treatment (Fig. 2B). In contrast, Tur in intraspecific treatment had significantly higher weight gain that those in interspecific and control treatments (Kruskal–Wallis chi-squared value = 10.01, df = 2, *p* = 0.006). Additionally, negative results for Pla in ex-situ conditions were due to considerable tissue loss of micro-colonies at week 12 as compared to the initial weight (Week 0). Intraspecific interaction for all three species cultured under in-situ conditions showed that only Pla had significantly higher weight gain (ANOVA, *F* (1,0.032) = 29.0, *p* = 9.62e−05) as compared to those under ex-situ conditions, whereas interspecific interaction for all three species cultured under in-situ conditions had significantly higher weight gain than micro-colonies in ex-situ conditions (Pla: ANOVA, *F* (1,0.078) = 69.09, *p* = 8.75e−07; Lit: ANOVA, *F* (1,0.004) = 6.27, *p* = 0.025; Tur: ANOVA, *F* (1,0.007) = 5.23, *p* = 0.038).

## Discussion

The significant noteworthy finding of the present study is that all three coral species, i.e., *Lithophyllon undulatum*, *Turbinaria mesenterina* and *Platygyra sinensis* are potentially suitable for mariculture and transplantation, albeit a longer culture period would be required to further support our present findings. Amongst the species tested, *L. undulatum* thrived best regardless of culture conditions and interactions, supported by results of both good survivorship and low percentage of tissue loss (Table 2, Fig. 1A,B). However, unlike *L. undulatum* and *T. mesenterina,* the survivorship of *P. sinensis* cultured in ex-situ conditions was affected by both culture conditions in particular water temperature and interactions as *P. sinensis* without interaction (the control treatment) survived well with no tissue loss (Fig. 1B) despite being grown in a suboptimal temperature environment. A previous thermal study showed that micro-colonies of *P. sinensis* thrived well at 28 °C as compared to those cultured at 30 °C, whilst micro-colonies of *T. mesenterina* were able to tolerate temperatures ranging from 28 to 32 °C (Loo, unpublished data). Conversely, *P. sinensis* had the highest growth rate at 30 °C as compared to 28 °C and their growth was higher than *T. mesenterina* when both species were cultured at 30 °C. This finding concurs with our growth results under in-situ conditions, where *P. sinensis* had significantly higher weight gain than *T. mesenterina* and *L. undulatum* (Fig. 2A). In the present study, the mean water temperature in the aquarium and field was recorded between 29.8 and 29.9 °C (Table 1). The same thermal study also demonstrated that massive corals such as *P. sinensis* performed poorly in response to elevated temperatures as compared to laminar corals such as *T. mesenterina* (Loo, unpublished data). As such, high variations in tissue loss and weight gain amongst coral species tested in both in-situ and ex-situ conditions can be partly attributed to water temperature and coral growth form. However, aggressive interaction between *T. mesenterina* and *P. sinensis* under in-situ interspecific treatment showed that *P. sinensis* is also a distinct possibility. Sweeper tentacles of *P. sinensis* could be induced to develop from normal feeding tentacles^30^ by spatial competition^31^ and these sweeper tentacles were indeed observed in the micro-colonies cultured in ex-situ conditions during the present study. During interspecific interaction, extracoelentric digestion is first exerted, followed by the interference of epifauna and eventually the development of sweeper tentacles by corals^32^. In a 1-year laboratory experiment, *Platygyra daedalea* colonies developed sweeper tentacles to attack adjacent *Favites complanata* colonies and damaged 55% of the latter’s soft tissue and caused 30% mortality of *F. complanata* colonies^33^. A similar interactive study also revealed that *P. daedalea* was able to dominate and secured space on reefs, at the expense of other corals in the Red Sea^31^. Additionally, a study involving paired interaction experiments between *P. sinensis, Platygyra pini* and different morphological variants of *P. daedalea* showed that *P. sinensis* was always dominant once competition had taken place. The results showed dead bands on dominated colonies^34^. Both *P. sinensis* and *P. daelalea* are ranked moderately aggressive in aggressiveness hierarchy^35^. These observations concur with our results of interspecies interaction under in-situ conditions, where *P. sinensis* was the dominant species with the highest mean weight gain, followed by *T. mesenterina*, with the weakest competitor being *L. undulatum* (Fig. 2A). Nonetheless, the results of interspecific interactions amongst coral species cannot be solely attributed to hierarchy and the development of sweeper tentacles. Position of contact, the presence of epifauna, colony size and environmental conditions^32^ all affect the outcomes of interspecific competition.

Micro-colonies of *T. mesenterina* in the aquarium were less affected by *P. sinensis* as *P. sinensis* did not survive well in ex-situ conditions. Different environmental conditions alter the outcome of spatial interaction between and within coral taxa. For instance, both *Pocillopora damicornis* and *Acropora hyacinthus* sustained substantial decreased growth rates when competing against other coral taxa as compared to that of in non-competing treatments^12^.

Unlike the well-studied mechanisms demonstrated by interspecific competition, little is known about the outcomes of non-clonal intraspecific interactions as most past studies have assumed that madreporarian corals do not generally display intraspecific aggression^21^. The interacting colonies of the same species that grow close to each other may either result in beneficial fusion^36^ or cessation of growth at the contact region^19^. Intraspecific interaction in hermatypic corals is an eminently complicated episode that could be of highly ecological paramount albeit it is harder to exemplify intraspecific interaction than interspecific interaction^37^ and interspecific competition is likely weaker than intraspecific competition^23^. Previous studies showed that the competitive consequences of two competing conspecific corals are the combination of at least four mechanisms of aggressiveness i.e., (a) benign morphological action involving neither abnormal alteration in the colony morphology and a “retreat growth” circumstance^19,21^ nor “filling” and “overgrowth” ^21,38^; (b) chemical action by means of allelochemicals where either the growth pattern of inferior colonies is impacted, leading to “retreat growth”^19,21^ or subordinate colonies are impaired by soluble chemicals from a distance^21^; (c) behavioural action through digestion of the subordinate colonies via extracoelentric feeding^38^ or discharge of nematocytes^21^; and (d) energetic-physiological mechanism where there is an oriented transfer of photosynthetic products from the inferior colonies to the superior competitors^39^ or the diminution of fecundity and growth rate^19^. Intraspecific contact interactions may also result in mutually beneficial interactions instead of competition. For instance, intraspecific interactions amongst *Agaricia tenuifolia* colonies on the turbulent Belize reefs resulted in the mutual strengthening of their skeletons and interdigitation^40^. The present study also demonstrated that all three coral species cultured in both in-situ and ex-situ conditions generally had higher survival and lower tissue loss than those in interspecific interaction (Table 2, Fig. 1A,B).

Intraspecific aggregation is plausibly beneficial to subordinate competitors through the reduction of the number and intensity of unfavourable competition occurrences^41^. Minimal heterotrophic interactions during the formation of conspecific aggregates favour weak competitors. Coupled with the reduction of intensity of intraspecific interference between highly competitive individuals and weak individuals, this enables the reservation of energy for interspecific competition^41^, which are more pervasive^42^. Aggregated spatial orientations encourage species coexistence in hermatypic corals^4,3^. Their coexistence can be sustained given that the overall fitness expenditure of corals for growth, competition and fecundity is lower than the energy acquired by an individual species^44^. Hence, a small alteration in growth rates provides additional advantages for coral survivorship and persistence^45^.

Selection of suitable micro-colony sizes for transplantation is of great importance for competitive success of sedentary organisms like corals, as larger colonies had been demonstrated to display greater competitive advantages^46^. Small coral colonies must avoid being overtopped and overgrown by other sessile organisms, and also not impaired by incidental grazers^43^. Conversely, large corals are unlikely to encounter complete mortality caused by coral competitors and predators. This study demonstrated that 3 cm × 3 cm coral micro-colonies are suitable for transplantation, as none of the micro-colonies sustained total mortality throughout the 3-month experiment (Table 2, Fig. 1A,B). Similarly, medium-size transplants of *Porites lutea* (3.5 cm × 3.5 cm) had significantly higher survivorship than the small-size transplants (2.5 cm × 2.5 cm) and grew at a similar rate as the large-size transplants (5.0 cm × 5.0 cm)^47^. In a separate transplantation study, the survival of 3–4 cm colonies of *Echinopora lamellosa* and *Merulina scabricula* was higher than larger colonies of 6–7 cm in length and width^48^. As such, the use of medium size-coral fragments for transplantation is not only suitable for optimum coral growth and survival but also can prevent further overexploitation of the wild corals and mitigate negative impacts on the donor colonies. Coral morphologies such as foliose, massive, encrusting and branching forms also influence the survivorship and growth of coral transplants. For instance, branching corals from the families Acroporidae and Pocilloporidae tend to grow faster and only require minimum maintenance compared to massive and encrusting corals such as Poritidae and Faviidae^49^. However, branching corals are typically short-lived as they are more susceptible to environmental perturbations than other coral morphologies^50^. Sole reliance on such corals for restoration could reduce the biodiversity in rehabilitated areas^48^. In this study, high survival rates of laminar and massive corals in in-situ conditions (Table 2) are indicative of their suitability for transplantation.

The essential life-preserving resources i.e., sugars, amino acids, small peptides and carbohydrates are generally limiting in the marine environment. They are in high demand to support their somatic growth, reproduction and maintenance^51^ of corals. In addition, these sessile organisms also invest energy to develop defensive mechanisms including the use of mesenterial filaments and sweeper tentacles^11^ and also allelopathy^52^. Interaction amongst hermatypic corals involves the regeneration of colonies, repair of tissue and formation of agonistic features applied to attack neighbours^30,53^. The results of weight gain of coral species (Fig. 2A,B) demonstrated that interactions did not affect growth rate greatly, and minimal energy was expended. As such, they are likely to coexist with their competitors in the local environments^43^. Conversely, different environmental conditions had significant impacts on the fitness of coral micro-colonies tested. Ex-situ cultivation might not be necessary for transplantation of fragmented corals as our present results showed that coral micro-colonies cultured in in-situ conditions had higher survival and growth than those cultured in ex-situ conditions (Table 2, and Fig. 2A,B). A previous study has also demonstrated that the survivorship and growth of one-year nursery-reared and wild *Echinopora lamellosa* and *Merulina scabricula* were comparable after transplanting them in the west of Lucero, Bolinao, Pangasinan, Philippines^48^. These results contradict current transplantation techniques that require the coral micro-colonies to be kept in ex-situ conditions prior to transplanting them to seawalls^6^. Instead, fragmenting the coral colonies on site and directly transplanting these micro-colonies may be a better way to increase the successful rate of coral gardening and rehabilitation. Such an approach is also cost-effective. Transportation of nursery-cultured corals back to the natural reef is the priciest component of the coral gardening protocol amounting to approximately 50% of the entire project expenditure, as compared to the costs of construction and maintenance of coral nurseries, which accounted for 5% and 10% per year, respectively^54^. Nonetheless, confirmatory studies are needed to investigate the effectiveness of transplantation with and without ex-situ nursery intervention. The effects of size and age of micro-colonies at time of transfer from ex-situ nursery to seawalls on their survivorship, growth and health status should also be enumerated for different species.

## Conclusions

The performance of *Lithophyllon undulatum* micro-colonies was neither affected by intra- and interspecific interactions as well as culture conditions. In contrast, micro-colonies of *Platygyra sinensis* were relatively sensitive to both environmental perturbations and interactions, and *Turbinaria mesenterina* was susceptible to interspecific interaction. Nevertheless, we suggest that these three coral species are potentially suitable to be transplanted onto seawalls, as these coral micro-colonies thrived well in in-situ conditions after only two weeks of acclimatization in an ex-situ nursery. We foresee that fragmented coral micro-colonies could possibly be directly transplanted to reduce potential loss of colonies during ex-situ cultivation, as farmed coral micro-colonies require time to adapt and acclimatize in a new environment which may result in mortality. Our present study also showed that *L. undulatum* can be transplanted together with *P. sinensis* and *T. mesenterina*, but the latter two coral species should not be transplanted close to each other. Transplantation of these coral micro-colonies at the same in-situ experimental site is underway for a long-term growth and survivorship monitoring.

## Materials and methods

### Site description

**Figure 3:**
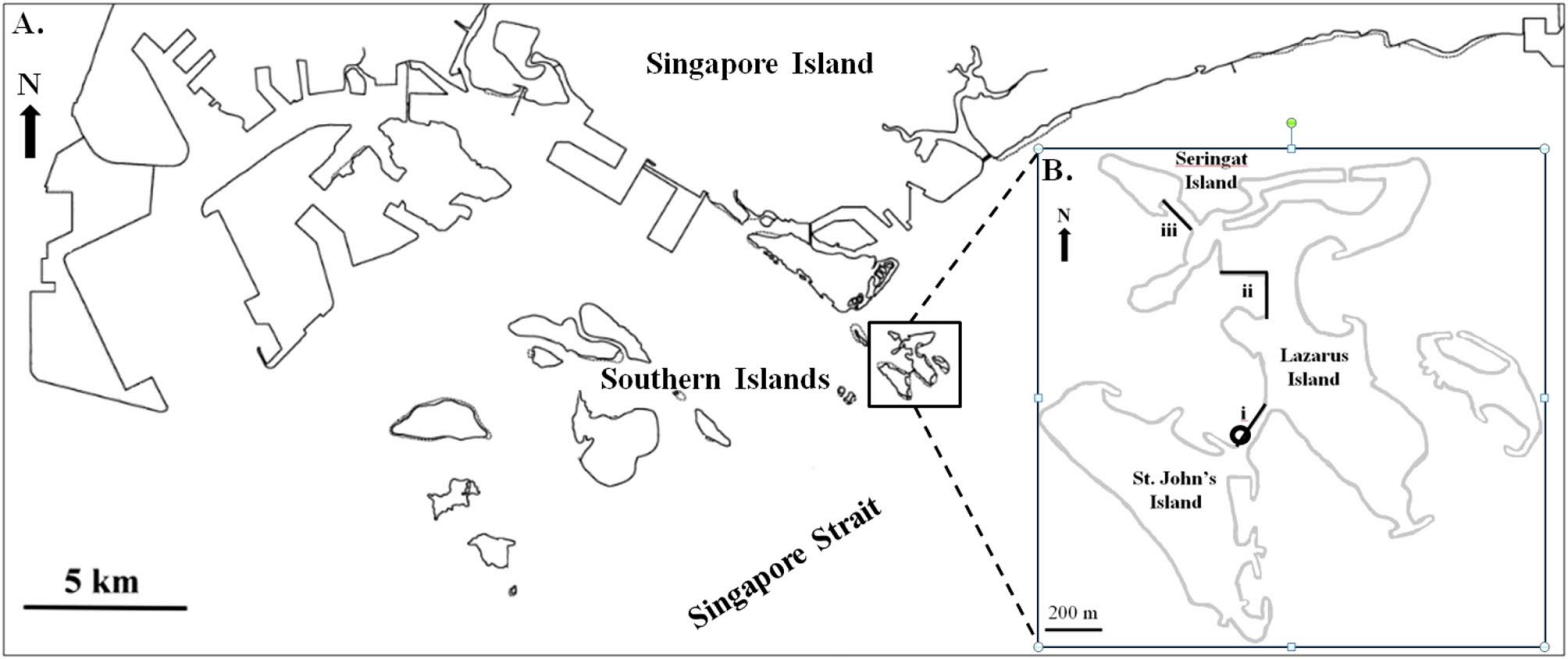
Map (**A**) shows the southern coastline of Singapore Island and the outlying Southern Islands in the Singapore Strait; Map (**B**) indicates the location of seawall sites examined i.e., (i) St. John’s Island (SJI)-Lazarus Causeway (N 1° 13′ 17.8′′ E 103° 51′ 05.7′′), (ii) Lazarus Island (N 1° 13′ 40.1′′ E 103° 51′ 05.7′′) and (iii) Seringat Island (N 1° 13′ 48.3′′ E 103° 50′ 55.8′′), the length of seawall surveyed (black straight line) and the in-situ experimental site (circled in black). Both maps were generated from Google Maps in which Map (**A**) was traced by hand, whilst Map (**B**) was extracted directly from Google Maps and modified using Microsoft PowerPoint 2010 and Paint software.

This study was carried out at St. John’s Island and Lazarus Island in the Singapore Strait. These two islands were chosen because of their richness and diversity of coral species^55^, easily accessible sloping seawalls and close proximity to the St. John’s Island National Marine Laboratory (SJINML) where the ex-situ experiment was conducted. Several seawalls of St. John’s Island (SJI)-Lazarus Causeway (N 1° 13′ 17.8′′ E 103° 51′ 05.7′′), Lazarus Island (N 1° 13′ 40.1′′ E 103° 51′ 05.7′′) and Seringat Island (N 1° 13′4 8.3′′ E 103° 50′ 55.8′′) were surveyed by snorkelling during low tide to determine potential coral species for interaction study, sample collection and also to evaluate a suitable seawall site for conducting in-situ experiments. The length of seawall surveyed at SJI-Lazarus causeway, Lazarus Island and Seringat Island was 230 m, 375 m and 190 m, respectively (Fig. 3).

### Species selection

Based on Singapore’s coral distribution^4,55^ and our recent seawall survey for corals, *Lithophyllon undulatum, Turbinaria mesenterina* and *Platygyra sinensis* were selected based on abundance, growth form and colour. Firstly, *P. sinensis* was a dominant species on seawalls, whilst *T. mesenterina* was least common, and *L. undulatum* was rare on seawalls. Secondly, those of *T. mesenterina* and *L. undulatum* were in the form of flat laminae, in contrast to *P. sinensis* which was massive growth form. Transplanting the said species together could provide different micro environments to enhance habitat complexity on seawalls. Thirdly, these three coral species had different colours and shapes, especially *L. undulatum* which exhibited a striking green colouration and white margin around corallites and transplantation of these species on seawalls could help to create an aesthetically pleasing coastline. These coral species were collected under the research permit NP/RP17-037 issued by National Parks Board (NParks), Singapore.

### Experimental design

Parent colonies of *L. undulatum, T. mesenterina* and *P. sinensis* from the intertidal seawalls of Lazarus Island were acclimated in an outdoor flow-through tank at SJINML for one week before being fragmented into 48 micro-colonies each 3 cm × 3 cm in size using a hammer and chisel so to ensure that the colonies were not too small to affect survival but still had adequate number of replicates^56^. These fragmented micro-colonies were kept for another week in the same tank to allow them to recover after which they were subjected to five treatments with four replicates for each treatment i.e., Treatment 1: In-situ intraspecific interaction (n = 8 micro-colonies for each species); Treatment 2: In-situ interspecific interaction (n = 8); Treatment 3: Ex-situ intraspecific interaction (n = 8); Treatment 4: Ex-situ interspecific interaction (n = 8) and Treatment 5: Ex-situ individual micro-colonies experienced no interaction (control, n = 16). Each treatment comprised four replicates.

In this study, the effects of coral-coral interaction on (1) coral survivorship, (2) per cent tissue loss and (3) growth were evaluated. Coral micro-colonies were spaced equidistant from each other approximately 1 cm apart for interaction to take place but no direct contact, so that the micro-colonies could still grow^32^. For intraspecific treatments where a replicate consisted of two micro-colonies of each coral species tested, a 6-cm gap was maintained between these three species to prevent interaction.

Treatments 1 to 4 were conducted by attaching coral micro-colonies on small mesh netting using marine epoxy, whilst the micro-colonies in Treatment 5 were attached to individual tiles using ethyl cyanoacrylate gel. Marine epoxy was used to elevate the flat micro-colonies so that the micro-colonies of different species were at the same height for interaction to take place. For Treatments 1 and 2, the micro-colonies on small mesh netting were respectively attached on two large mesh nettings prior to securing them on a sloping seawall of SJI-Lazarus Causeway (N 1° 13′ 17.8′′ E 103° 51′ 05.7′′) (Fig. 3) at the intertidal zone approximately 0.3 m above chart datum. This was to ensure that the coral micro-colonies were submerged in water most of the time, but still accessible during low tides. Additionally, the purpose of attaching the coral micro-colonies on mesh nettings instead of directly transplanting them onto the rocky seawalls to study coral-coral interaction was to eliminate the possible impact of existing organisms on the early stage of micro-colonies in in-situ environments. For Treatments 3, 4 and 5, micro-colonies were kept in outdoor flow-through tanks at SJINML. Treatments 3 and 4 were cultured in a tank with 80 cm × 80 cm and 40 cm depth of water, whilst Treatment 5 was cultured in a long tank with 80 cm × 170 cm and water depth of 40 cm, depending on the availability of culture tanks. They were sheltered from direct sunlight using a black mesh frame, together with wave generators (model: Tunze 6045) to increase water flow in the tanks.

### Data collection

The effect of intraspecific and interspecific interactions in terms of survivorship was determined by examining the living tissue at the edges of two coral micro-colonies placed 1 cm apart for tissue loss after 12 weeks. Healthy micro-colonies were recorded as (+ 1), whereas unhealthy micro-colonies were recorded as (0). Percent tissue loss of coral micro-colonies were determined at week 0 and week 12 based on the digital images obtained using a digital camera (Canon S100) and images were analysed using ImageJ. Growth of coral micro-colonies was determined based on their weight in seawater using the culture water in which they were grown and measured using a weighing balance (Mettler Toledo ME4002). Environmental parameters were recorded weekly, i.e., salinity, temperature, DO and pH were measured using a HQ40D portable multi meter. HOBO pendant temperature and light data loggers were deployed in both in-situ and ex-situ environments.

### Statistical analyses

The probability of survivorship of each coral species tested was calculated by dividing the number of living coral micro-colonies with the number of replicates. Mean growth and percentage of tissue loss of coral micro-colonies that were subjected to treatments i.e., intra- and interspecific interactions and in-situ and ex-situ conditions were compared using software R version 3.4.3. Additionally, water quality in both culture environments i.e., salinity, temperature, DO and pH was analysed individually. Levene’s Test was used to determine the homogeneity of data sets prior to performing either one-way ANOVA or Kruskal Wallis Test.

## Data Availability

All data analysed for this study are presented in this articles and the raw data are available from the corresponding author upon request.
